# Expression Patterns of DLL3 across Neuroendocrine and Non-neuroendocrine Neoplasms Reveal Broad Opportunities for Therapeutic Targeting

**DOI:** 10.1158/2767-9764.CRC-24-0501

**Published:** 2025-02-14

**Authors:** John R. Lozada, Andrew Elliott, Mark G. Evans, James Wacker, Kathleen M. Storey, Emily A. Egusa, Nicholas A. Zorko, Akhilesh Kumar, Anthony Crymes, Elisabeth I. Heath, Benedito A. Carneiro, Heloisa P. Soares, Frank Cichocki, Jeffrey S. Miller, Emil Lou, Himisha Beltran, Emmanuel S. Antonarakis, Charles J. Ryan, Justin H. Hwang

**Affiliations:** 1 Division of Hematology, Oncology, and Transplantation, University of Minnesota-Twin Cities, Minneapolis, Minnesota.; 2 Masonic Cancer Center, University of Minnesota-Twin Cities, Minneapolis, Minnesota.; 3 Medical Scientist Training Program (MD/PhD), University of Minnesota, Minneapolis, Minnesota.; 4 Caris Life Sciences, Phoenix, Arizona.; 5 Department of Medicine, Keck School of Medicine of the University of Southern California, Los Angeles, California.; 6 Department of Oncology, Wayne State University Karmanos Cancer Institute, Detroit, Michigan.; 7 Legorreta Cancer Center, Brown University, Providence, Rhode Island.; 8 Huntsman Cancer Institute, University of Utah, Salt Lake City, Utah.; 9 Dana-Farber Cancer Institute, Boston, Massachusetts.

## Abstract

Neuroendocrine neoplasms (NEN) encompass a diverse set of malignancies with limited precision therapy options. Recently, therapies targeting DLL3 have shown clinical efficacy in aggressive NENs, including small cell lung cancers and neuroendocrine prostate cancers. Given the continued development and expansion of DLL3-targeted therapies, we sought to characterize the expression of DLL3 and identify its clinical and molecular correlates across diverse neuroendocrine and non-neuroendocrine cancers. Here, we interrogated paired DNA and RNA-sequencing from 1,589 NENs across 29 sites, as well as 203,252 tumors across 47 cancer types. We found that high transcriptomic levels of *DLL3* correlated with more aggressive histologic and mutational patterns in NENs, with adverse survival outcomes being reflected in NENs originating from the lung, pancreas, stomach, and small bowel. The heterogeneity in *DLL3* expression across NENs was largely explained by site of origin, with lung, prostate, and bladder NENs exhibiting relatively high levels of *DLL3*, whereas gastroenteropancreatic NENs displayed relatively low expression levels. Although the therapeutic targeting of DLL3 may be less applicable for gastroenteropancreatic NENs, we did find an upregulation of alternative targets such as *SEZ6*, *CELSR3*, and *SSTR2* in these settings. Lastly, expanding our investigation into non-neuroendocrine cancers, we detected an enrichment of *DLL3* in both low-grade and high-grade gliomas, Merkel cell carcinomas, medulloblastomas, and melanomas, with such enrichment being associated with prolonged overall survival in gliomas, but worse overall survival in melanomas. Altogether, we demonstrate that DLL3 represents an attractive target for subsets of neuroendocrine and non-neuroendocrine cancers and uncover opportunities for future therapeutic strategies.

**Significance:**

DLL3-targeted therapies have recently shown robust clinical efficacy in aggressive neuroendocrine cancers, positioning them to fulfill a great unmet need in these settings. Here, we examine the clinical and biological correlates of DLL3 expression in both neuroendocrine and non-neuroendocrine cancers. Our findings may stimulate the development and application of DLL3-targeted therapies, as well as other precision therapies, in neuroendocrine cancers and beyond.

## Introduction

Neuroendocrine neoplasms (NEN) represent a heterogeneous group of cancers sharing overlapping histologic features with non-neoplastic neuroendocrine cells and the production of neurosecretory proteins (1–3). Although NENs can arise at most anatomic sites, they exhibit distinct biological characteristics and varying degrees of clinical aggressiveness. This is particularly exemplified when classifying NENs into the two histologic subdivisions set forth by the World Health Organization—well-differentiated neuroendocrine tumors (NET) and poorly differentiated neuroendocrine carcinomas (NECs; refs. 2–4). NETs display relatively indolent clinical behavior, whereas NECs represent aggressive diseases with poor outcomes. Nevertheless, for both NETs and NECs, therapeutic options beyond conventional modalities (i.e., chemotherapy, surgical resection, and radiotherapy) remain limited.

Given the aggressive nature of several NEN subsets, particularly small cell lung cancers (SCLC) and treatment-emergent neuroendocrine prostate cancers (NEPC), substantial efforts have focused on elucidating and exploiting the molecular alterations underlying NEN pathogenesis. Previous studies have shown that NECs are commonly underpinned by recurrent genomic alterations in *TP53*, *RB1*, and *PTEN*, whereas NETs are commonly underpinned by alterations in *MEN1*, *DAXX*, *CDKN2A*, and *ATRX* (3, 5, 6). These alterations, in conjunction with upregulation of the achaete-scute complex-like 1/achaete-scute homolog 1 (ASCL1) transcription factor, drive neuroendocrine lineage specification in both SCLC and NEPC (7–9). ASCL1 acts to induce a transcriptional program that promotes and supports a neuronal and stem cell fate while repressing an epithelial fate through the downregulation of Notch signaling (7–9). Common transcriptional targets of ASCL1 include neuroendocrine markers, such as *INSM1*, and Notch ligands *DLL1* and *DLL3*. Notably, DLL3 serves as an inhibitory Notch ligand, with a few studies suggesting a role for DLL3 in the growth and metastatic potential of neuroendocrine cells (10, 11).

DLL3 has been of particular interest as a precision therapeutic target for NENs given its upregulation in multiple NENs but not in normal adult tissues (12). The field has had vested interests in the development of antibody–drug conjugates (13, 14), antibody radioconjugates (15), bispecific T-cell engagers (BiTE; refs. 16–18), and chimeric antigen receptor T-cell therapies (19, 20). Indeed, the engagement of DLL3 on NENs has shown therapeutic promise with phase II clinical trials of the DLL3/CD3 BiTE, tarlatamab, reporting objective responses in 40% of patients with previously treated SCLC (21). Of note, tarlatamab, received FDA approval in May 2024 for use in patients with chemotherapy-refractory, extensive-stage SCLC. Given this recent advance, DLL3-targeted therapies are poised to fill a great unmet need in SCLC and other aggressive NENs.

Here, we aggregated a cohort of 1,589 NENs across 29 anatomic sites and explored the landscape of *DLL3* expression to identify clinical, genomic, and biological correlates across diverse NEN settings. Our analyses demonstrated that NENs from several anatomic sites, particularly the lung, prostate, and bladder, exhibit high *DLL3* expression and that *DLL3* is associated with high-grade NENs. We also explored the expression patterns of *DLL3* and other promising precision therapy targets to identify alternative strategies in *DLL3*-low settings. Lastly, we also extended our investigation into >200,000 tumor samples across 47 cancer types, finding upregulated expression in gliomas, Merkel cell carcinomas, and melanomas. We envision our findings may inform the further development and applications of DLL3-targeted therapies, as well as alternative therapeutic strategies for NENs.

## Materials and Methods

### Specimens

We retrospectively examined genomic alterations and corresponding survival outcomes for 203,252 patient tumor biopsies across 47 cancer types. This cohort included 1,589 NEN samples across 29 anatomic sites (Table 1). Comprehensive molecular profiling [DNA and RNA-sequencing (RNA-seq)] was performed in a Clinical Laboratory Improvement Amendments/College of American Pathologists/ISO15189-certified clinical laboratory (Caris Life Sciences). This study was carried out using retrospective de-identified clinical data, in accordance with 45 CFR 46.101(b; ref. 4) and the guidelines set forth by the Declaration of Helsinki, Belmont Report, and U.S. Common Rule. Thus, this study was deemed exempt at each institutional review board.

**Table 1:** Demographics of NEN samples by anatomic site

Cancer type	Median *DLL3* (TPM)	*N* total	*N* w/0 TPM *DLL3*	% w/0 TPM *DLL3*	*N* w/*DLL3*-high	% w/*DLL3*-high	Male	Female	Median age	Primary	Metastasis
Skin	8.998175	2	0.0	0.00	2	100	1	1	63.5	0	2
Kidney	7.348750	2	0.0	0.00	2	100	2	0	69.5	1	1
Anal	6.323540	17	0.0	0.00	14	82.35	5	12	63	10	7
Prostate	6.105810	66	1.0	1.52	50	75.76	66	0	69	23	43
Abdomen	5.960443	2	0.0	0.00	1	50	1	1	50.5	1	1
Lung	5.400185	122	2.0	1.64	87	71.31	42	80	68	48	74
Bile duct	5.374540	27	0.0	0.00	20	74.07	18	9	66	13	14
Bladder	4.691435	66	1.0	1.52	51	77.27	47	19	69.5	48	18
Esophagus	4.251460	32	0.0	0.00	22	68.75	25	7	67.5	21	11
Head and neck	3.859390	43	1.0	2.33	31	72.09	28	15	60	21	22
Gynecologic organ	3.609865	118	1.0	0.85	90	76.27	0	118	57.5	58	60
Colorectal	2.353520	141	8.0	5.67	80	56.74	74	67	60	73	68
Unknown primary	1.853830	294	12.0	4.08	161	54.76	169	125	66	NA	NA
Thymus	1.833560	9	0.0	0.00	5	55.56	6	3	54	1	8
Thyroid	1.726960	4	0.0	0.00	3	75	1	3	57	2	2
Unclear/other	0.843033	25	4.0	16.00	9	36	15	10	61	13	12
Ileocecal junction	0.820018	11	1.0	9.09	4	36.36	6	5	70	4	7
Pancreas	0.772417	232	10.0	4.31	85	36.64	136	96	62	71	161
Breast	0.770587	11	0.0	0.00	3	27.27	0	11	51	6	5
Liver	0.662014	13	1.0	7.69	5	38.46	6	7	75	11	2
Bone	0.571393	3	0.0	0.00	1	33.33	1	2	65	3	0
Appendix	0.478279	16	1.0	6.25	4	25	7	9	54	9	7
Stomach	0.347639	38	2.0	5.26	12	31.58	27	11	62	18	20
Gastrointestinal tract	0.238579	30	5.0	16.67	12	40	14	16	65.5	0	30
Peritoneum/retroperitoneum	0.232515	6	0.0	0.00	0	0	4	2	55	4	2
Adrenal gland	0.220075	89	7.0	7.87	11	12.36	37	52	50	43	46
Carotid body	0.217635	4	0.0	0.00	0	0	1	3	40	1	3
Small bowel	0.134410	150	14.0	9.33	12	8	79	71	64	62	88
Nervous system	0.099808	16	2.0	12.50	3	18.75	10	6	49	1	15

### Survival analysis

Real-world evidence outcomes were assessed using insurance claim data. Overall survival was defined using the date of treatment initiation to either (i) the date of death or (ii) date of last contact specified in the insurance claim repository. Patient death was assumed for any patient lacking a claim for more than 100 days as this holds true for more than 95% of patients with a recorded death in the National Death Index. Cox proportional hazard ratios were calculated for each group compared and significance was calculated using the log-rank statistic, with a *q* value cutoff of <0.05. Schoenfeld residuals were also tested to assess the suitability of Cox proportional hazard ratios.

### DNA next-generation sequencing

Genomic DNA was isolated from formalin-fixed, paraffin-embedded (FFPE) tumor samples that were microdissected for tumor purity. Samples were analyzed using the Illumina Nextseq (592-gene panel) or Novaseq 6000 (whole-exome) sequencers. For the 592-gene panel, a hybrid pulldown of baits was used to enrich for genes of interest at a high coverage and high read-depth. For whole-exome sequencing, a panel designed to enrich for >20,000 genes at a lower depth was used, which included a 500 Mb SNP backbone panel (Agilent Technologies) to aid in detecting gene amplifications and deletions.

### Whole-transcriptome sequencing

The Qiagen RNA FFPE tissue extraction kit was used to extract RNA from FFPE tumor biopsies. The Agilent TapeStation (RRID:SCR_019547) was used for RNA quality and quantity. Biotinylated RNA baits were hybridized to the corresponding synthesized and purified cDNA targets. The bait–target complexes were amplified using a post-capture PCR reaction. The whole transcriptome was sequenced to an average of 60 million reads using the Illumina NovaSeq 6500. Raw data was demultiplexed by the Illumina Dragen Bio-IT accelerator, trimmed, counted, and removed of PCR duplicates, and aligned to the human reference genome hg19 by the STAR aligner. Transcripts per million (TPM) molecules were calculated using the Salmon expression. *DLL3*-high status was defined globally using a threshold derived from 10× median expression of adenocarcinomas (ADC) of *DLL3* from ADCs of the lung and prostate (1.38 TPM).

### Tumor mutation burden

Tumor mutation burden (TMB) was measured by counting all non-synonymous mutations (missense, nonsense, in-frame insertion/deletion, and frameshift) found per tumor that had not yet been described as a germline alteration in dbSNP151, Genome Aggregation Database, or benign variants noted by Caris geneticists. A cutoff point of ≥10 mutations per megabase was used to define TMB-high status, based on the KEYNOTE-158 pembrolizumab trial.

### Immune cell deconvolution

To examine potential associations with unique tumor immune landscapes, whole-transcriptome sequencing data was subjected to immune cell deconvolution. quanTIseq was used to computationally infer immune cell fractions reliably in bulk RNA-seq data (22).

### Dependency analysis

Genetic dependencies in neuroendocrine cancer cell lines were accessed through the Cancer Dependency Map (DepMap) and based on pooled genome-scale short hairpin RNA (DEMETER2) or clustered regularly interspaced short palidromic repeat (CRISPR) screens (DepMap Public 24Q2; ref. 23). Cell lines with noted neuroendocrine features were selected using the Cell Line Selector tool. Dependencies were defined based on a gene effect threshold of < −0.5.

### Statistical analyses

Statistical significance was determined using *χ*^2^ and Mann–Whitney U tests with Benjamini–Hochberg corrections for multiple comparisons when appropriate.

### Data availability

This study did not generate any new raw DNA/RNA-seq data but rather used existing data from the Caris Life Sciences database through a formal letter of intent and subsequent data use agreement. Here, we researched the de-identified data collected in a real-world health care setting, and this is subject to controlled access for privacy and proprietary reasons. When possible, derived data supporting the findings of this study have been made available within the paper and its Supplementary Figures/Tables. Other data can be acquired through a letter of intent to Caris Life Sciences (https://www.carislifesciences.com/letter-of-intent/). Additional inquiries can be sent to Andrew Elliott at aelliott@carisls.com.

## Results

### High *DLL3* expression is associated with prototypical high-grade neuroendocrine features

**Figure 1:**
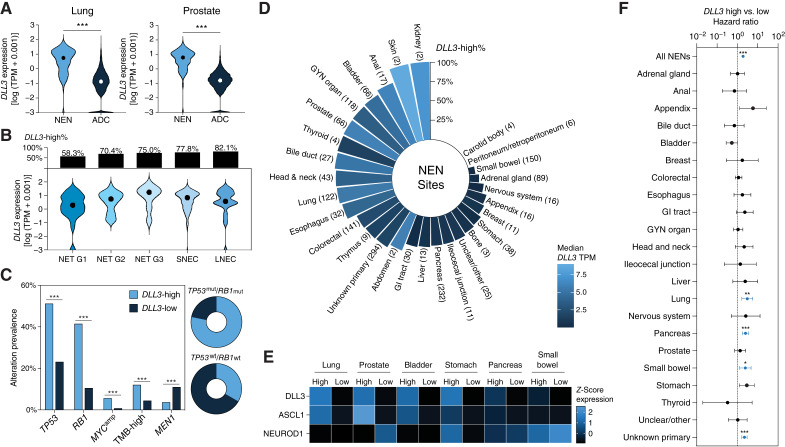
Expression patterns of *DLL3* across NENs. **A,** Violin plots displaying expression of *DLL3* in NENs and ADCs originating from the lung or prostate. **B,** Violin plots displaying expression of *DLL3* in lung NENs by histologic grade. Bar plots above show proportion of histologic grade that was defined as *DLL3*-high. LNEC, large cell neuroendocrine carcinoma; SNEC, small cell neuroendocrine carcinoma. **C,** Differences in genetic alterations between *DLL3*-high and -low NENs, as well as breakdown of samples with concurrent mutations or wildtype status in *TP53* and *RB1* by *DLL3*-high or -low NENs. **D,** Circular bar plot displaying proportion of *DLL3*-high samples by NEN anatomic site with sample sizes shown in parentheses. Bar color represents median *DLL3* expression for the site. **E,** Heatmap displaying relative expression of *DLL3*, *ASCL1*, and *NEUROD1* in *DLL3*-high and -low NENs from the lung, prostate, bladder, stomach, pancreas, and small bowel. **F,** Forest plot showing association between *DLL3* expression level and real-world overall survival. Dotted line represents the hazard ratio of 1.0. *, *q* < 0.05; **, *q* < 0.01; ***, *q* < 0.001.

As significant efforts have been placed on targeting aggressive NENs of the lung and prostate, we first examined the expression of *DLL3* in NENs of these sites (Fig. 1A). In doing so, we observed significantly higher expression of *DLL3* in NENs derived from both the lung and prostate compared with site-matched adenocarcinomas (ADCs). Considering the minimal expression of *DLL3* in prostate ADCs and lung ADCs, we define *DLL3*-high as 10× the median TPM of prostate and lung ADCs for our further analyses.

Given the potential heterogeneity of NEN lesions in anatomic sites, we subjected the lung and prostate NENs to retrospective histologic analysis based on the World Health Organization criteria for NETs/NECs (2, 4). Indeed, our cohort of lung NENs comprised a spectrum ranging from grade 1 NETs to large cell NECs, in stark comparison to our prostate NENs which were primarily small cell NECs. It should be noted, however, that the prostate NENs we examined constituted histologically pure (non-focal) neuroendocrine lesions, in contrast to more frequently observed lesions with mixed ADC and neuroendocrine elements (24). Stratifying lung NENs by histologic annotation, we identified increased expression of *DLL3* across lung NET grades, and increased proportions of *DLL3*-high samples throughout, with 58.3% of grade 1 lung NETs being defined as *DLL3*-high in comparison to 82.1% of large cell NECs (Fig. 1B).

We then asked whether *DLL3* expression correlates with certain mutational features underpinning NENs. Using our global thresholds for defining *DLL3*-high status across all NENs in the aggregate, we found that *DLL3*-high NENs exhibit increased rates of mutations involving *TP53* (51.3% vs. 23.2%; *q* < 0.001) and *RB1* (41.5% vs. 10.6%; *q* < 0.001), as well as *MYC* amplifications (5.7% vs. 0.9%; *q* < 0.001) and high TMB (12.1% vs. 4.5%; *q* < 0.001; Fig. 1C). Conversely, *DLL3*-low NENs harbored more mutations in *MEN1* (3.8% vs. 11.1%; *q* < 0.001), which are prevalent in low-grade NENs. In line with these patterns, we also identified an enrichment of *TP53*^*mut*^/*RB1*^*mut*^ NENs in *DLL3*-high samples, whereas most *TP53*^*wt*^/*RB1*^*wt*^ NENs were *DLL3*-low.

### *DLL3* is differentially expressed across anatomic sites and is commonly associated with adverse outcomes

Considering the vastly different nature of NENs across anatomic sites, we next expanded our investigation of *DLL3* expression to a cohort of 1,589 NENs across 27 anatomic sites (Table 1). We detected high levels of *DLL3* expression in both prostate and lung NENs, alongside those originating from the bladder. Using our threshold for *DLL3*-high status, we found that the majority of NENs in the prostate (76%), lungs (71%), and bladder (77%) displayed high expression of *DLL3* at the transcriptomic level (Fig. 1D). In stark contrast, the majority of gastroenteropancreatic (GEP)-NENs had relatively low levels of *DLL3*, with a minority of NENs from the pancreas (37%), stomach (32%), and small bowel (8%) being defined as *DLL3*-high. Further corroborating such heterogeneity in *DLL3* expression across anatomic sites, we find that unbiased clustering of the NEN samples using the top 1,000 variably expressed genes led to discernible separation by *DLL3* expression level and anatomic site (Supplementary Fig. S1).

To parse out the associations we observed in *DLL3* expression across anatomic sites, we examined the relative expression levels of *DLL3* with the canonical neuroendocrine transcription factor, *ASCL1*, and non-canonical transcription factor, *NEUROD1*. Across the six sites examined, *DLL3* expression strongly correlated with the expression of *ASCL1* (Fig. 1E; Supplementary Fig. S2), corroborating the regulation of *DLL3* expression by ASCL1 (7). In contrast, *DLL3* expression was not strongly correlated with *NEUROD1* expression in NENs, with the exception in lung NENs (Supplementary Fig. S2). Interestingly, in the GEP-NENs, we observed relatively low levels of *ASCL1* that coincided with relatively higher levels of *NEUROD1*, potentially pointing to different molecular mechanisms underlying their pathogenesis (Fig. 1E).

Given our previous findings associating high *DLL3* expression with higher grade neuroendocrine features, we investigated the relevance of *DLL3* expression across sites with real-world outcomes. We found high *DLL3* expression to be associated with worse overall survival across all NENs in the aggregate [HR, 1.96; 95% confidence interval (CI), 1.74–2.23; *q* < 0.001; Fig. 1F; Supplementary Table S1]. This was further exemplified when stratifying NENs by site of origin, with high *DLL3* expression being associated with adverse outcomes in NENs derived from the lung, pancreas, and small bowel. High *DLL3* expression was also associated with worse overall survival in NENs with unknown primaries (HR, 2.23; 95% CI, 1.65–3.02; *q* < 0.001).

### *DLL3*-high NENs display altered immune landscapes

**Figure 2:**
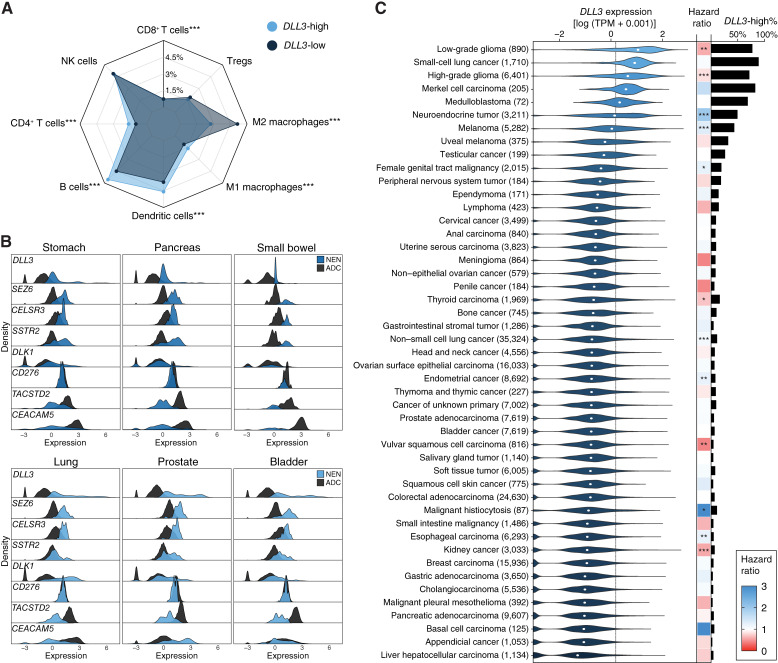
Correlates of *DLL3* expression with immune repertoire, alternative targets, and non-neuroendocrine cancers. **A,** Radar plots displaying differences in immune infiltrates between *DLL3*-high and -low NENs. **B,** Ridgeline plots comparing the expression density of *DLL3* and alternative precision targets in NENs and site-matched ADCs. **C,** Violin plots, bar plots, and heatmap displaying expression of *DLL3*, percent *DLL3*-high, and hazard ratio, respectively, across 47 cancer types. Dotted line depicts threshold for *DLL3*-high status. *, *P* < 0.05; **, *P* < 0.01; ***, *P* < 0.001.

The current landscape of DLL3-targeted therapies is primarily comprised of immune-based targeting modalities, with BiTEs, antibody–drug conjugates, or antibody-radiotherapy combinations. As such, we sought to understand whether NENs with high expression of *DLL3* exhibit altered profiles of immune infiltrates compared with those with low expression of *DLL3*. Employing an immune deconvolution tool to estimate fractions of distinct immune cell types in bulk RNA-seq data, we found that both *DLL3*-high and -low NENs had relatively low levels of CD8^+^ T cell infiltration compared with other immune cell types (Fig. 2A). In *DLL3*-high NENs, NK cells and B cells constituted the primary immune infiltrates, whereas NK cells and pro-tumor M2 macrophage populations were the most abundant in *DLL3*-low NENs. The stark abundance of NK cells is notable considering their association with prolonged survival in NENs. Comparing the immune repertoires of *DLL3*-high versus -low NENs, high *DLL3* expression was associated with an enrichment of M1 macrophages (1.60% vs. 1.09%; *q* < 0.001), B cells (5.56% vs. 4.49%; *q* < 0.001), dendritic cells (4.58% vs. 3.72%; *q* < 0.001), and CD4^+^ T cells (1.64% vs. 0.99%; *q* < 0.001), and depletion of M2 macrophages (2.74% vs. 5.14%; *q* < 0.001). These patterns were also relatively conserved at a finer granularity, when we examined site-specific and histology-specific settings (Supplementary Fig. S3). For instance, lung NETs with high *DLL3* expression harbored statistically increased proportions of B cells (7.21% vs. 6.04%; *q* = 0.032), dendritic cells (7.97% vs. 5.11%; *q* = 0.002), and M1 macrophages (1.80% vs. 1.03%; *q* = 0.009), with a relative depletion in M2 macrophages compared with *DLL3*-low lung NETs (2.64% vs. 4.41%; *q* = 0.003).

### SEZ6, CELSR3, and SSTR2 represent alternative precision targets for GEP-NENs

As we found *DLL3* expression to be relatively low in GEP-NENs, specifically those originating from the stomach, pancreas, and small bowel, we aimed to identify alternative precision targets that may be promising in settings in which *DLL3* is less applicable. Thus, we compared the expression of *DLL3* to that of other targets under investigation in diverse NEN settings—*SEZ6*, *CELSR3*, *SSTR2*, *DLK1*, *CD276*, *TACSTD2*, and *CEACAM5* (Fig. 2B). We also further compared expression levels to site-matched ADCs to identify specificity towards NENs. The expression patterns of the alternative targets were relatively consistent across the GEP-NEN sites, with stomach, pancreatic, and small bowel NENs displaying relatively uniform upregulation of *SEZ6*, *CELSR3*, and *SSTR2* compared with their ADC counterparts. The expression of these three genes was also discernibly higher than that of *DLL3* in these settings. Notably, *SEZ6* and *CELSR3* were also upregulated in most lung, prostate, and bladder NENs, with a subset of them also displaying upregulation of *SSTR2*. In contrast, *DLK1* was only upregulated in lung NENs and a subset of prostate NENs, whereas *CEACAM5* was only upregulated in prostate NENs. *CD276*, on the other hand, displayed relatively high, uniform expression in both NENs and ADCs across all six sites, corroborating its potential as a pan-cancer target. Additionally, we leveraged the genetic depletion screens from the DepMap (23) to identify other vulnerabilities in cancer cell lines with neuroendocrine phenotypes. From these analyses, we found that *DLL3* did not constitute a genetic dependency in neuroendocrine cancer cell lines (Supplementary Fig. S4). We did, however, identify *MYBL2* as a potential dependency, in line with its role in driving phenotypic plasticity in NEPCs (25).

### *DLL3* is upregulated in subsets of non-neuroendocrine cancers

Lastly, we explored the potential utility of DLL3 as a precision therapy target in cancer types outside of NENs. Aggregating a cohort of 203,252 tumor samples across 47 cancer types, we found upregulation of *DLL3* in the majority of low-grade gliomas (78%), high-grade gliomas (72%), Merkel cell carcinomas (83%), medulloblastomas (69%), and melanomas (43%), in conjunction with SCLCs (89%) and NETs (49%; Fig. 2C). Additionally, we identified small subsets of *DLL3*-high tumors in other cancer types, including kidney cancers (7%), thyroid carcinomas (16%), and non-SCLCs (11%). Interestingly, in contrast to the relatively consistent associations we observed between high *DLL3* expression and poor overall survival across NENs, we found differential patterns with real-world outcomes across different cancer types (Fig. 2C; Supplementary Table S2). High *DLL3* expression was associated with prolonged survival in brain tumors, such as low-grade gliomas (HR, 0.61; 95% CI, 0.46–0.81; *P* < 0.001) and high-grade gliomas (HR, 0.85; 95% CI, 0.80–0.91; *P* < 0.001), as well as in kidney cancers (HR, 0.60; 95% CI, 0.47–0.75; *P* < 0.001) and thyroid carcinomas (HR, 0.72; 95% CI, 0.58–0.90; *P* = 0.004). On the other hand, high *DLL3* expression was associated with poorer prognoses in melanomas (HR, 1.32; 95% CI, 1.22–1.44; *P* < 0.001), non-SCLCs (HR, 1.13; 95% CI, 1.09–1.18; *P* < 0.001), and female genital tract malignancies (HR, 1.24; 95% CI, 1.07–1.44; *P* = 0.004).

## Discussion

In this study, we investigated the expression patterns of *DLL3* across diverse sites for neuroendocrine and non-NENs. In doing so, we demonstrate that DLL3-targeted therapies may be best focused on lung, prostate, and bladder NENs given their relatively high expression levels of *DLL3* compared with stomach, pancreatic, and small bowel NENs that displayed relatively low transcriptomic expression of *DLL3*. Nonetheless, alternative precision therapy targets such as *SEZ6* and *CELSR3* should be considered for NENs in which the targeting of DLL3 is less viable. Interestingly, high *DLL3* expression was also associated with higher grade NEN features as reflected through histologic grade, mutational repertoire, and clinical outcomes. Finally, we demonstrate the potential expanded utility of DLL3-targeted therapies beyond NENs, finding robust expression of *DLL3* in gliomas, medulloblastomas, and melanomas, among other non-neuroendocrine cancer types.

The role of DLL3 expression level in predicting response to DLL3-directed therapies remains an area of continued investigation. Preclinical studies have shown that tarlatamab is capable of eliciting antitumor T cell responses in both DLL3-high and -low NEPC models, but that loss of DLL3 expression may serve as a mechanism of resistance (24). Moreover, exploratory analyses from the phase I clinical trial of tarlatamab (NCT03319940) in recurrent SCLC suggest that higher DLL3 expression may be predictive of greater clinical benefit (16). Findings from phase I studies of tarlatamab in NEPCs have also shown improved response rates in patients with DLL3-expressing NEPC (26). In line with these, the phase I clinical trial for the DLL3-targeted antibody–drug conjugate, rovalpituzumab tesirine, showed that patients with high DLL3 expression exhibited greater overall response rates than patients with low DLL3 expression (35% vs. 0%, respectively; ref. 13). However, subsequent studies testing rovalpituzumab tesirine did not demonstrate similar responses in patients with DLL3-high disease, and further clinical investigation was discontinued owing to inferior overall survival compared with standard-of-care chemotherapy alongside high toxicity profiles (27). As DLL3-directed therapies become more prominent in the clinical setting, further work is warranted to identify biomarkers predictive of therapeutic response.

To the best of our knowledge, this study represents the largest investigation of *DLL3* across both neuroendocrine and non-NENs. As such, we also aimed to reconcile previously published studies examining the level of DLL3 expression in single anatomic sites for NENs. We confirmed trends in DLL3 expression that have been reported previously in which 75% of prostate NENs in our cohort exhibit high *DLL3* expression, matching previous reports of 76% of NEPCs representing DLL3-high lesions (28). Conversely, we found that only 31% of stomach and 8% of small bowel NENs were classified as *DLL3*-high, resembling previous findings of similarly low prevalences of 29% and 22%, respectively (29). The spectrum of *DLL3* expression in our cohort, in conjunction with what has been previously reported, emphasizes the site-specific nature of NENs. Whether this is more reflective of a divergent underlying biological mechanism or is due to tissue-specific microenvironmental factors leaves much to be speculated. It is worth noting, however, the associations we found with high *DLL3* expression and the prevalence of genomic alterations associated with NECs versus NETs, particularly considering the propensity of the latter with GEP-NENs versus most prostate and bladder NENs representing carcinomas.

Further exploring the association between *DLL3* expression and the anatomic site of origin, we find a rather consistent trend of tumors derived from the neural crest, such as gliomas and melanomas representing cancers with relatively robust *DLL3* expression. Although the embryonic origin of NEN remains unresolved, current evidence points to derivation from the neural crest or the endoderm being rather site-specific (30). Neuroendocrine cells of the lung, thymus, and thyroid are considered to exhibit the most “neural-like” features, whereas neuroendocrine cells of the digestive tract and pancreas are less so, exhibiting more “epithelial-like” features (30). In line with high *DLL3* expression potentially marking more (neuro)endocrine features, we found a strong association between the level of *DLL3* expression and that of the canonical neuroendocrine transcription factor *ASCL1*, which underlies the prominence of *DLL3* expression in lung, prostate, and bladder NENs. Further corroborating these patterns, however, are the associations we found with high *DLL3* and better overall survival in low-grade and high-grade gliomas. In gliomas, ASCL1 activates neuronal differentiation pathways and suppresses tumorigenicity (31). This is in stark contrast to prostate cancer in which ASCL1 promotes lineage plasticity and treatment resistance (9). Although we must reiterate that our analyses remain exploratory in nature, our findings nonetheless provide provocative parallels between the underlying pathogenesis of NENs and other cancer types. Additionally, the exact role of DLL3 in NEN pathogenesis remains unclear, with a paucity of studies exploring the functional consequences of DLL3 expression and the lack of DLL3-directed inhibitors.

Given that most DLL3-targeted therapies in development focus on harnessing T-cell immunity, we hoped to explore how the intratumoral immune repertoire may be associated with the level of *DLL3* expression. We found that *DLL3*-high NENs harbor a greater abundance of “immune-supportive” populations of B cells, dendritic cells, M1 macrophages, and CD4^+^ T cells with a reciprocal lower abundance of “immune-suppressive” M2 macrophage and regulatory T cell populations. This suggests that *DLL3*-high NENs may be more amenable to immunotherapeutic agents given a more permissive microenvironment. Although little differences were observed in effector populations, we were intrigued by the substantially greater infiltration of NK cells compared with CD8^+^ T cells in NENs. We previously found that NEPCs were enriched with NK cells compared with other types of prostate cancer, with the infiltration of CD56^dim^ NK cells being associated with better overall survival (32). Altogether, these findings suggest that NK cell–based therapies may potentially serve as promising alternative strategies for targeting NENs, particularly as a number of NK cell–based approaches are currently in development (33–35).

Expanding out studies beyond DLL3-targeting agents, we explored the potential utility of engaging alternative precision therapy targets in settings in which DLL3 expression may be low or absent, focusing largely on therapeutic targets already in clinical development. In doing so, we identified SEZ6 and CELSR3 as viable targets across diverse NEN settings, as well SSTR2 serving as a promising target for GEP-NENs. Notably, a SEZ6-targeted antibody–drug conjugate demonstrated clinical efficacy in a phase I trial for patients with advanced SCLC and NENs (NCT05599984; ref. 36) as did an SSTR2-targeting radiopharmaceutical in a phase Ib trial for GEP-NENs (NCT05477576; ref. 37). On the other hand, CELSR3 has only recently been identified as a target for NEPCs, with a CELSR3/CD3 BiTE showing efficacy in preclinical mouse models of NEPC (38). Additionally, we found rather consistent and uniform upregulation of *CD276* (B7-H3) in both ADCs and NENs across sites, further positioning B7-H3 as a versatile precision target across cancer. Altogether, these point to alternative therapeutic directions for NENs that should be considered alongside DLL3-targeted therapies.

Our study has several limitations. Although we performed in-depth histologic reviews of NENs from the lung and prostate, the representation of NEN histologic grades in other sites was not investigated. Further studies are warranted to determine whether differences in histologic grade may underlie the expression patterns of *DLL3* seen in other anatomic sites, particularly as the propensity of NETs versus NECs differs by site. We also based our findings on transcriptomic expression of *DLL3*, which may not necessarily translate to protein expression or membrane localization. Regardless, we must note that our findings match previously reported analyses of DLL3 expression defined by immunohistochemistry and that prior studies in NEPCs showed concordance between transcriptomic and protein expression of DLL3. Lastly, we defined our threshold for *DLL3*-high status using lung and prostate ADCs as a baseline for minimal expression. Although our threshold again recapitulated previous reports of DLL3 expression, it remains unknown what level of expression may be required for therapeutic benefit.

In conclusion, by exploring the clinical and biological correlates of *DLL3* expression, we reveal the expanded utility of DLL3-targeted therapies across diverse neuroendocrine and non-neuroendocrine cancers. We also propose novel avenues for the development of future therapeutic strategies that involve the engagement of alternative precision targets in settings in which DLL3 may be less applicable. We envision that our findings will open opportunities to greatly expand the application of DLL3-targeted therapies and stimulate the development of therapeutic strategies for NENs.

## Supplementary Material

Table S1 DLL3-high versus -low hazard ratios across NEN anatomic sites

Table S2 DLL3-high versus -low hazard ratios across cancer.

Figure S1 Supplementary Figure S1: UMAP view of NEN samples across diverse anatomic sites. Samples were unbiasedly clustered based off the top 1,000 variably expressed genes across samples and annotated using (A) DLL3 expression level and (B) anatomic site of origin.

Figure S2 Supplementary Figure S2: Correlations between expression of DLL3, ASCL1, and NEUROD1 across select NEN sites. Scatterplots displaying the expression of DLL3 vs ASCL1 (top) and DLL3 vs NEUROD1 (bottom) across NEN sites. Expression is depicted as log(TPM + 0.001). Spearman correlations and corresponding p-values are shown for each site.

Figure S3 Supplementary Figure S3: Immune repertoire of DLL3-high versus –low NENs across anatomic sites. Stacked radar plots comparing the imputed cell fractions of immune cells between DLL3-high and -low samples in (A) lung NECs, (B) lung NETs, (C) prostate NENs, and (D) bladder NENs. *q < 0.05, **q < 0.01

Figure S4 Supplementary Figure S4: Genetic dependencies in neuroendocrine cancer cell lines. Scatterplot displaying gene depletion effects in NEN lines represented in DepMap. Data points represent the mean gene depletion effect of each gene target. Highlighted genes represent potential dependencies (gene effect < -0.5).
