# Supplementary material for: Expression Patterns of DLL3 across Neuroendocrine and Non-neuroendocrine Neoplasms Reveal Broad Opportunities for Therapeutic Targeting
Source: Cancer Res Commun. 2025 Feb 14;5(2):318–26. doi: 10.1158/2767-9764.CRC-24-0501 (PMC11827001; doi:10.1158/2767-9764.CRC-24-0501)
Supplement: Table S1 — DLL3-high versus -low hazard ratios across NEN anatomic sites [file crc-24-0501_table_s1_suppst1.pdf]

Supplementary Table S1: DLL3-high versus -low hazard ratios across NEN anatomic sites.

Primary Tumor Site	Hazard Ratio	HR Lower 95% CI	HR Upper 95% CI	P-value	Q-value
Adrenal gland	1.02	0.46	2.26	0.9551	0.9551
Anal	0.72	0.18	2.81	0.6326	0.7732
Appendix	5.83	1.28	26.62	0.0228	0.0836
Bile Duct	0.71	0.23	2.13	0.5387	0.7079
Bladder	0.53	0.28	0.99	0.0472	0.1483
Breast	1.74	0.29	10.48	0.547	0.7079
Colorectal	1.14	0.78	1.67	0.4975	0.7079
Esophagus	1.77	0.68	4.64	0.2436	0.4613
GI Tract, NOS	2.34	0.91	6.00	0.0764	0.2101
GYN Organ	1.08	0.63	1.85	0.7897	0.8687
Head and Neck	2.16	0.80	5.83	0.1282	0.3134
Ileocecal junction	1.39	0.23	8.49	0.7227	0.8368
Liver	2.43	0.60	9.89	0.2161	0.4613
Lung	3.07	1.65	5.69	0.0004	0.0029
Nervous system	2.58	0.51	13.02	0.2516	0.4613
Pancreas	2.51	1.79	3.52	<0.0001	<0.0001
Prostate	1.36	0.73	2.54	0.3306	0.5595
Small Bowel	2.46	1.28	4.73	0.0069	0.0380
Stomach	2.92	1.25	6.87	0.0137	0.0603
Thyroid	0.33	0.02	5.33	0.4373	0.6872
Unclear/Other	1.04	0.36	3.02	0.9424	0.9551
Unknown Primary	2.23	1.65	3.02	<0.0001	<0.0001
