# Supplementary material for: Expression Patterns of DLL3 across Neuroendocrine and Non-neuroendocrine Neoplasms Reveal Broad Opportunities for Therapeutic Targeting
Source: Cancer Res Commun. 2025 Feb 14;5(2):318–26. doi: 10.1158/2767-9764.CRC-24-0501 (PMC11827001; doi:10.1158/2767-9764.CRC-24-0501)
Supplement: Table S2 — DLL3-high versus -low hazard ratios across cancer. [file crc-24-0501_table_s2_suppst2.pdf]

Supplementary Table S2: DLL3-high versus -low hazard ratios across cancer.

Cancer Type	Hazard Ratio	HR Lower 95% CI	HR Upper 95% CI	P-value	Q-value
Anal Carcinoma	1.01	0.71	1.43	0.9776	0.9954
Appendiceal Cancer	0.82	0.46	1.46	0.5068	0.6806
Basal Cell Skin Cancer	3.17	1.11	9.02	0.0306	0.0959
Bladder Cancer	0.99	0.88	1.12	0.884	0.9662
Bone Cancer	1.15	0.82	1.62	0.4186	0.5962
Breast Carcinoma	1.08	0.98	1.19	0.1264	0.2829
Cancer of Unknown Primary	1.04	0.94	1.15	0.4091	0.5962
Cervical Cancer	1.03	0.87	1.21	0.7522	0.8623
Cholangiocarcinoma	1.08	0.92	1.27	0.3423	0.5363
Colorectal Adenocarcinoma	0.99	0.93	1.07	0.8736	0.9662
Endometrial Carcinoma	1.23	1.09	1.39	0.0011	0.0074
Ependymoma	1.19	0.56	2.54	0.6446	0.8188
Esophageal Carcinoma	1.23	1.08	1.40	0.0021	0.0099
Female Genital Tract Malignancy	1.24	1.07	1.44	0.0036	0.0141
Gastric Adenocarcinoma	1.24	1.00	1.54	0.0501	0.1472
Gastrointestinal Stromal Tumors	1.23	0.79	1.91	0.3636	0.5513
Head and Neck Cancers	0.92	0.79	1.07	0.2765	0.4762
High Grade Glioma	0.85	0.80	0.91	<0.0001	<0.0001
Kidney Cancer	0.60	0.47	0.75	<0.0001	<0.0001
Liver Hepatocellular Carcinoma	0.76	0.53	1.09	0.1374	0.2935
Low Grade Glioma	0.61	0.46	0.81	0.0006	0.0047
Lymphoma	0.64	0.38	1.10	0.1056	0.2641
Malignant Histiocytosis	5.29	1.76	15.91	0.003	0.0128
Malignant Pleural Mesothelioma	0.64	0.30	1.36	0.2495	0.4691
Medulloblastoma	1.00	0.43	2.32	0.9954	0.9954
Melanoma	1.32	1.22	1.44	<0.0001	<0.0001
Meningioma	0.43	0.25	0.73	0.0019	0.0099
Merkel Cell Carcinoma	1.60	0.85	3.02	0.1471	0.3006
Neuroendocrine Tumor	1.98	1.74	2.25	<0.0001	<0.0001
Non-small Cell Lung Cancer	1.13	1.09	1.18	<0.0001	<0.0001
Non-Epithelial Ovarian Cancer	1.10	0.65	1.88	0.7175	0.8615
Ovarian Surface Epithelial Carcinoma	1.09	0.99	1.20	0.0711	0.1966
Pancreatic Adenocarcinoma	0.94	0.85	1.05	0.2626	0.4747
Penile Cancer	0.44	0.16	1.20	0.1098	0.2641
Peripheral Nervous System Tumor	0.82	0.32	2.09	0.6792	0.8401

Prostatic Adenocarcinoma	1.05	0.91	1.21	0.493	0.6806
Salivary Gland Tumor	1.00	0.66	1.51	0.9886	0.9954
Small Cell Lung Cancer	1.05	0.87	1.27	0.6304	0.8188
Small Intestinal Malignancy	0.65	0.44	0.95	0.0268	0.0900
Soft Tissue Tumors	1.09	0.96	1.25	0.1695	0.3319
Squamous Cell Skin Cancer	1.32	0.80	2.18	0.2837	0.4762
Testicular Cancer	1.00	0.66	1.52	0.9855	0.9954
Thymoma & Thymic Cancer	0.89	0.44	1.78	0.7332	0.8615
Thyroid Carcinoma	0.72	0.58	0.90	0.0042	0.0152
Uterine Serous Carcinoma	1.13	0.97	1.33	0.1124	0.2641
Uveal Melanoma	0.86	0.64	1.16	0.3358	0.5363
Vulvar Squamous Cell Carcinoma	0.43	0.25	0.74	0.002	0.0099
