# Supplementary material for: Expression Patterns of DLL3 across Neuroendocrine and Non-neuroendocrine Neoplasms Reveal Broad Opportunities for Therapeutic Targeting
Source: Cancer Res Commun. 2025 Feb 14;5(2):318–26. doi: 10.1158/2767-9764.CRC-24-0501 (PMC11827001; doi:10.1158/2767-9764.CRC-24-0501)
Supplement: Figure S1 — Supplementary Figure S1: UMAP view of NEN samples across diverse anatomic sites. Samples were unbiasedly clustered based off the top 1,000 variably expressed genes across samples and annotated using (A) DLL3 expression level and (B) anatomic site of origin. [file crc-24-0501_figure_s1_suppsf1.pdf]

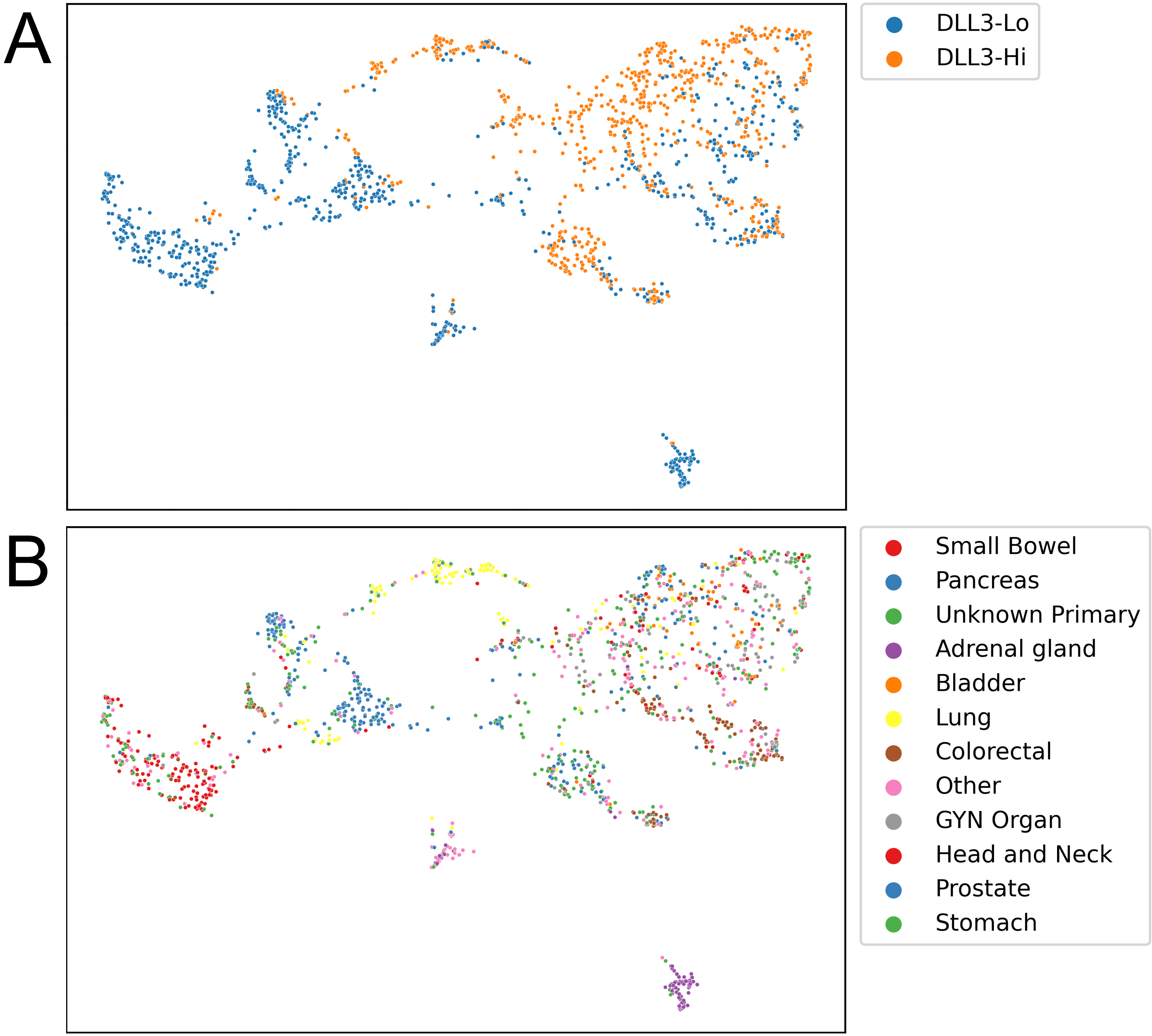

Supplementary Figure S1: UMAP view of NEN samples across diverse anatomic sites. Samples were unbiasedly clustered based off the top 1000 variably expressed genes across samples and annotated using (A) *DLL3* expression level and (B) anatomic site of origin.
