# Supplementary material for: Expression Patterns of DLL3 across Neuroendocrine and Non-neuroendocrine Neoplasms Reveal Broad Opportunities for Therapeutic Targeting
Source: Cancer Res Commun. 2025 Feb 14;5(2):318–26. doi: 10.1158/2767-9764.CRC-24-0501 (PMC11827001; doi:10.1158/2767-9764.CRC-24-0501)
Supplement: Figure S2 — Supplementary Figure S2: Correlations between expression of DLL3, ASCL1, and NEUROD1 across select NEN sites. Scatterplots displaying the expression of DLL3 vs ASCL1 (top) and DLL3 vs NEUROD1 (bottom) across NEN sites. Expression is depicted as log(TPM + 0.001). Spearman correlations and corresponding p-values are shown for each site. [file crc-24-0501_figure_s2_suppsf2.pdf]

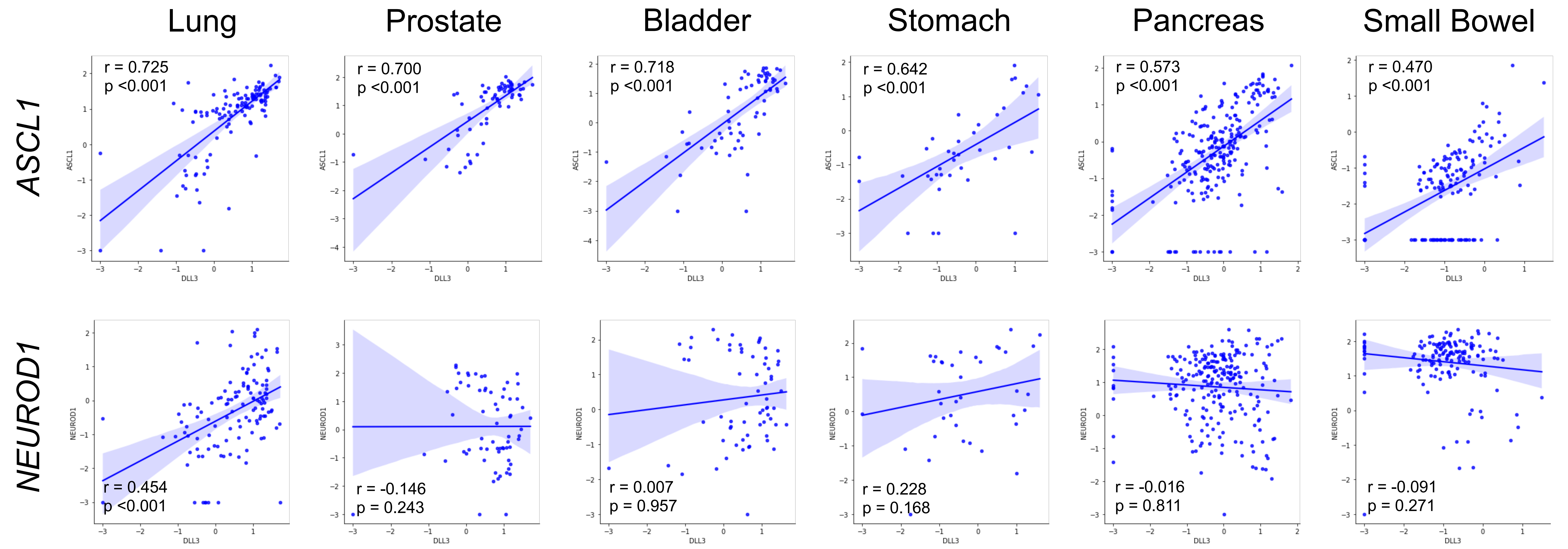

Supplementary Figure S2: Correlations between expression of DLL3, ASCL1, and NEUROD1 across select NEN sites. Scatterplots displaying the expression of DLL3 vs ASCL1 (top) and DLL3 vs NEUROD1 (bottom) across NEN sites. Expression is depicted as log(TPM + 0.001). Spearman correlations and corresponding p-values are shown for each site.
