# Supplementary material for: Expression Patterns of DLL3 across Neuroendocrine and Non-neuroendocrine Neoplasms Reveal Broad Opportunities for Therapeutic Targeting
Source: Cancer Res Commun. 2025 Feb 14;5(2):318–26. doi: 10.1158/2767-9764.CRC-24-0501 (PMC11827001; doi:10.1158/2767-9764.CRC-24-0501)
Supplement: Figure S3 — Supplementary Figure S3: Immune repertoire of DLL3-high versus –low NENs across anatomic sites. Stacked radar plots comparing the imputed cell fractions of immune cells between DLL3-high and -low samples in (A) lung NECs, (B) lung NETs, (C) prostate NENs, and (D) bladder NENs. *q < 0.05, **q < 0.01 [file crc-24-0501_figure_s3_suppsf3.pdf]

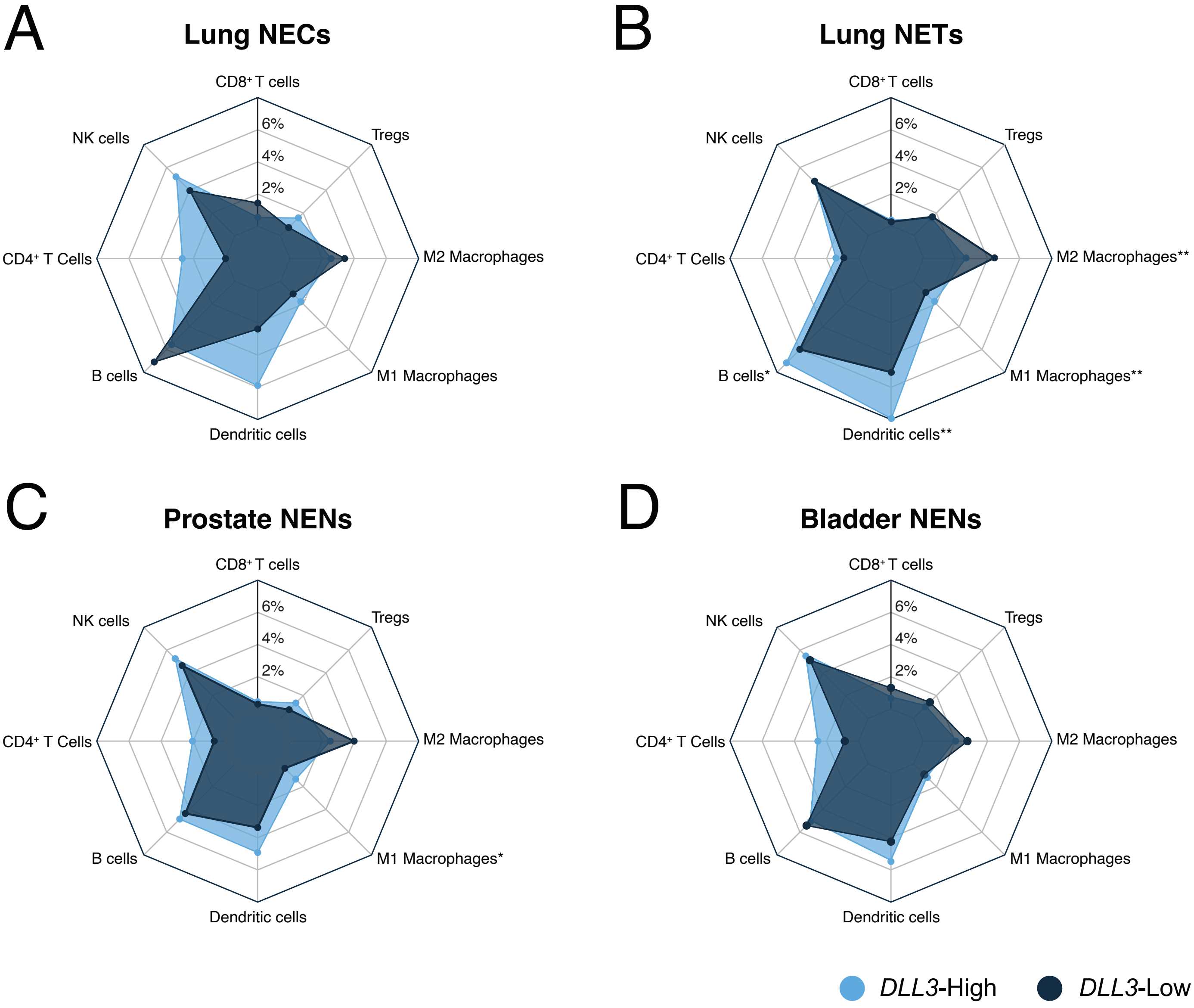

Supplementary Figure S3: Immune repertoire of DLL3-high versus –low NENs across anatomic sites. Stacked radar plots comparing the imputed cell fractions of immune cells between *DLL3*-high and -low samples in (A) lung NECs, (B) lung NETs, (C) prostate NENs, and (D) bladder NENs. *q<0.05, **q<0.01
