# Supplementary material for: Expression Patterns of DLL3 across Neuroendocrine and Non-neuroendocrine Neoplasms Reveal Broad Opportunities for Therapeutic Targeting
Source: Cancer Res Commun. 2025 Feb 14;5(2):318–26. doi: 10.1158/2767-9764.CRC-24-0501 (PMC11827001; doi:10.1158/2767-9764.CRC-24-0501)
Supplement: Figure S4 — Supplementary Figure S4: Genetic dependencies in neuroendocrine cancer cell lines. Scatterplot displaying gene depletion effects in NEN lines represented in DepMap. Data points represent the mean gene depletion effect of each gene target. Highlighted genes represent potential dependencies (gene effect < -0.5). [file crc-24-0501_figure_s4_suppsf4.pdf]

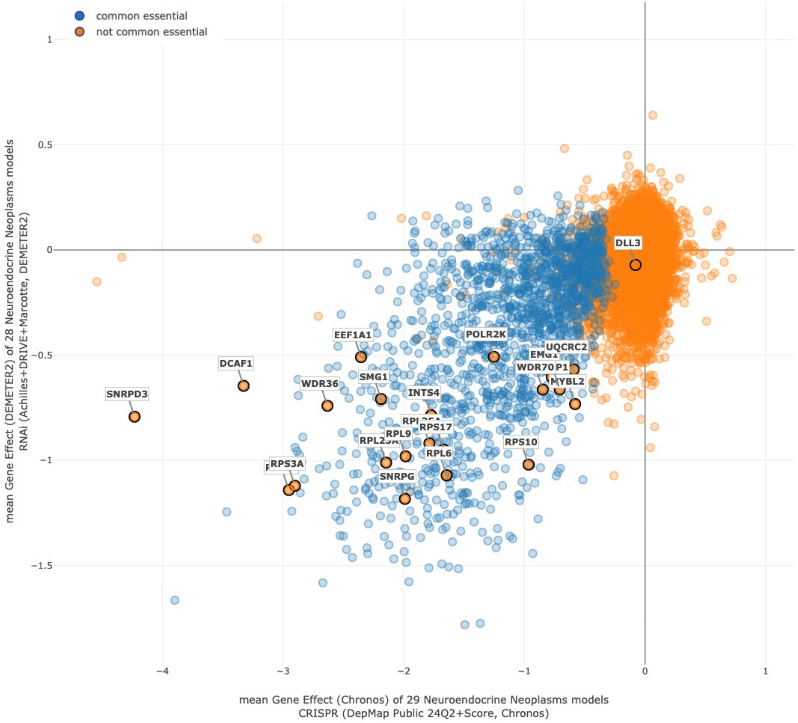

Supplementary Figure S4: Genetic dependencies in neuroendocrine cancer cell lines. Scatterplot displaying gene depletion effects in NEN lines represented in DepMap. Data points represent the mean gene depletion effect of each gene target. Highlighted genes represent potential dependencies (gene effect < -0.5).
